# Reversible cardiac function and left ventricular hypertrophy in a Chinese man with mitochondrial myopathy: a case report

**DOI:** 10.1186/s12872-023-03444-z

**Published:** 2023-09-15

**Authors:** Guiping Wu, Yijun Han, Lifeng Zhao, Hong Zhang, Xiuzhao Fan, Weiqin Li, Xiaowen Che, Yun Zhou

**Affiliations:** 1 Department of Cardiology, The Fifth Hospital of Shanxi Medical University (Shanxi Provincial People’s Hospital), Taiyuan, 030012 China; 2 Clinical Medical College, Jining Medical University, Jining, 272000 China; 3 Department of Microbiology Test, Taiyuan Center for Disease Control and Prevention, Taiyuan, 030012 China; 4 Department of Nephropathy, The Fifth Hospital of Shanxi Medical University (Shanxi Provincial People’s Hospital), Taiyuan, 030012 China; 5 Department of Ultrasound, The Fifth Hospital of Shanxi Medical University (Shanxi Provincial People’s Hospital), Taiyuan, 030012 China

**Keywords:** Mitochondrial myopathy, Heart failure, Respiratory failure, Pulmonary artery hypertension, Left ventricular hypertrophy

## Abstract

**Background:**

Mitochondrial myopathies (MMs) are a group of multi-system diseases caused by abnormalities in mitochondrial DNA (mtDNA) or mutations of nuclear DNA (nDNA). The diagnosis of mitochondrial myopathy (MM) is reliant on the combination of history and physical examination, muscle biopsy, histochemical studies, and next-generation sequencing. Patients with MMs have diverse clinical manifestations. In the contemporary literature, there is a paucity of reports on cardiac structure and function in this rare disease. We report a Chinese man with MM accompanied with both acute right heart failure and left ventricular hypertrophy.

**Case presentation:**

A 49-year-old man presented with clinical features suggestive of MM, i.e., ophthalmoparesis, weakness of the pharyngeal and extremity muscles, and respiratory muscles which gradually progressed to respiratory insufficiency. He had a family history of mitochondrial myopathy. He had increased levels of serum creatine kinase and lactate. Muscle biopsy of left lateral thigh revealed 8% ragged red fibers (RRF) and 42% COX-negative fibers. Gene sequencing revealed a novel heterozygote *TK2* variant (NM_001172644: c.584T>C, p.Leu195Pro) and another heterozygous variant (NM_004614.4:c.156+958G>A; rs1965661603) in the intron of *TK2* gene. Based on these findings, we diagnosed the patient as a case of MM. Echocardiography revealed right heart enlargement, pulmonary hypertension, left ventricular hypertrophy, and thickening of the main pulmonary artery and its branches. The patient received non-invasive ventilation and coenzyme Q10 (CoQ10). The cardiac structure and function were restored at 1-month follow-up.

**Conclusions:**

This is the first report of reversible cardiac function impairment and left ventricular hypertrophy in a case of adult-onset MM, nocturnal hypoxia is a potential mechanism for left ventricular hypertrophy in patients with MM.

## Background

Mitochondrial myopathies (MMs) are progressive muscle disorders caused primarily by mitochondrial dysfunction, including impairment of oxidative phosphorylation, damage of respiratory chain, and decreased maximum oxygen intake [1, 2]. MMs are caused by abnormalities in mitochondrial DNA (mtDNA) which are transmitted via X-linked, autosomal-recessive, and autosomal-dominant inheritance patterns [3]. The diagnosis of MM is reliant on the combination of history and physical examination, muscle biopsy, histochemical studies, and next-generation sequencing [4]. Two important diagnostic features of MMs are the presence of ragged-red fibers (RRF) and >5% cytochrome c oxidase (COX)-negative fibers. The whole exome screens are used to search for potential disease-causing genes.

MMs are more frequently seen in children and adolescents than adults. Patients with MM usually present symptoms of skeletal muscle fatigue, such as limb muscle weakness, ptosis, cramps, dyspnea, and even respiratory failure [5].

Here we report a case of MM which presented with reversible cardiac function impairment and left ventricular hypertrophy. The clinical course, auxiliary examination, and response to medical management during 1-year follow-up are described.

### Case presentation

**Fig. 1:**
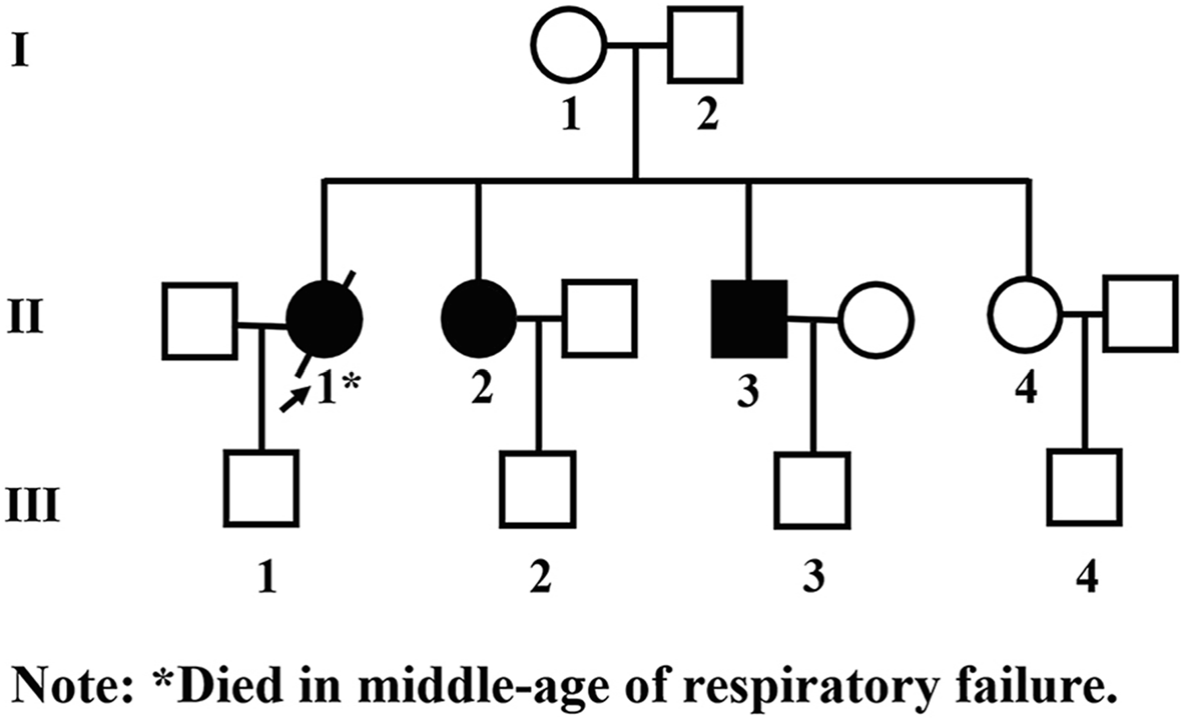
The pedigree of the patient: the index case is a family member, II-3.

The patient was a 49-year-old man from Shanxi province who was born to non-consanguineous parents. He presented with a history of progressive shortness of breath and edema of both lower limbs for 7 days. One week before admission, the patient developed shortness of breath in a supine position at night, which improved after 3-5 minutes of rest in the sitting position. The shortness of breath recurred 3–4 times per night, accompanied by progressive edema of both lower extremities. The patient denied any history of hypertension, diabetes, or smoking. Starting from the age of 8 years, the patient showed signs of bilateral upper and lower limb weakness during exercise and his sports tests never met the standard. At the age of 35 years, he started to complain of difficulty in swallowing solid food. Mild bilateral ptosis was noticed from the age of 45 years. He had a family history of MM. His eldest sister was diagnosed with MM based on histochemical analysis of skeletal muscle biopsy. However, no records of the exact site of mutation in the eldest sister were available. She developed respiratory failure at the age of 52 years and died of respiratory failure several years ago. The second sister showed progressive ptosis and bilateral limb weakness; however, she has not yet undergone skeletal muscle biopsy. The parents and the little sister of the patient appear in good health at present. The pedigree map of three generations of the family is shown in Fig. 1. The patient in our report is the family member, II-3.

**Fig. 2:**
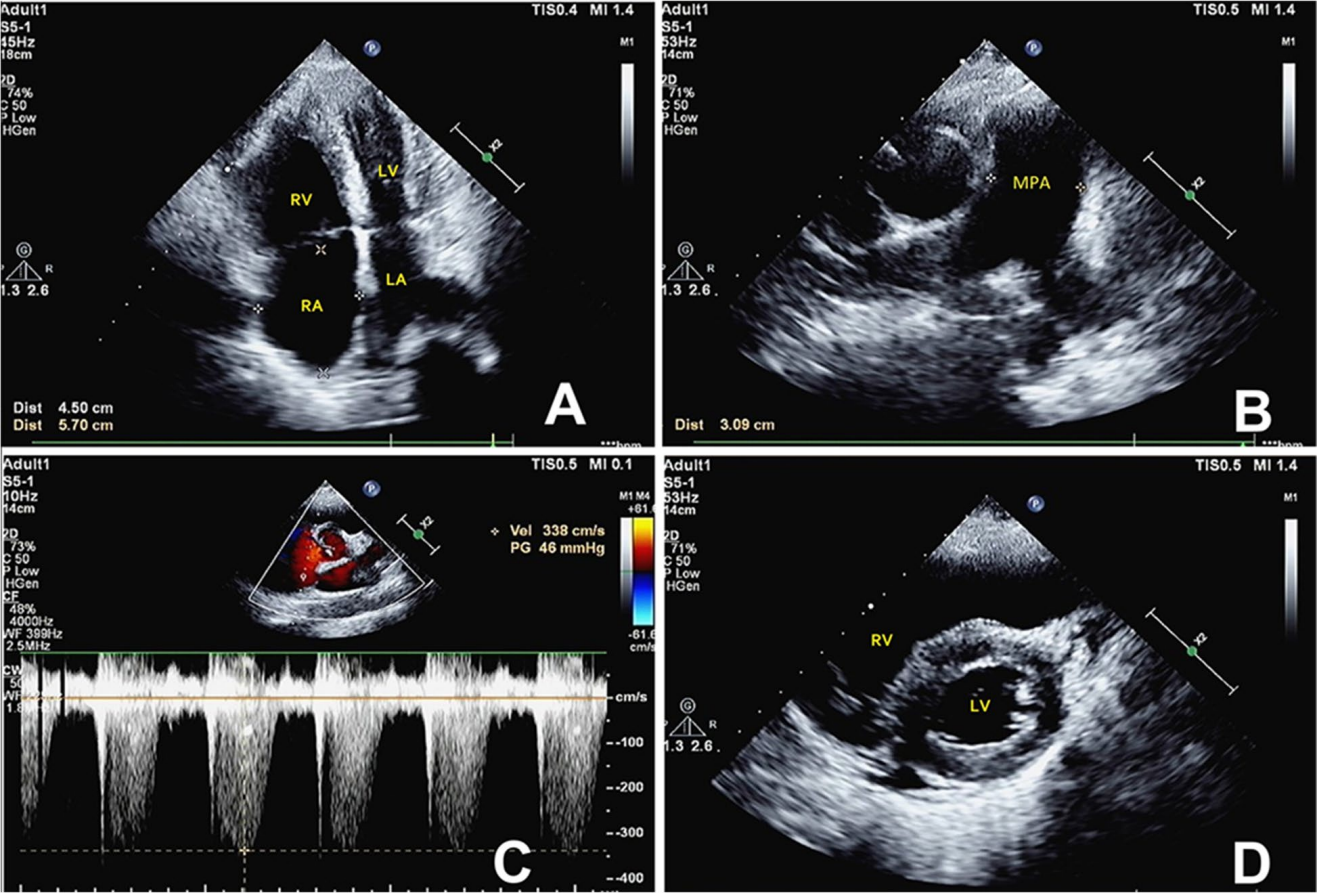
Pre-treatment echocardiography (**A**-**D**). Apical four-chamber view showing enlargement of right atrium (RA) and right ventricle (RV) (**A**); Short-axis view of the heart showing dilation of the main pulmonary artery (MPA) and its branches (**B**); Peak tricuspid regurgitation velocity by continuous Doppler, peak right ventricle–to–right atrial systolic pressure gradient is 51 mmHg (**C**); Left ventricle short-axial view showing symmetric slight thickening of the left ventricle (LV); LV end-diastolic wall thickness is 13 mm (**D**)

The patient had acute symptoms, but had normal sensorium at the time of hospitalization. He looked very thin and his height was 1.83 meters and weight was 49 kg. His vital signs were: body temperature, 36.6°C; pulse rate, 89 beats/min; respiratory rate, 25 beats/min; blood pressure, 124/85 mmHg. There was ptosis and facial weakness. Chest percussion showed dullness on the right chest wall and no significant enlargement of the heart; no cardiac murmur was heard on auscultation. Neurological examination showed no cranial nerve deficit. His cognitive abilities were normal. Ptosis was mild on the right and moderate on the left side. At rest, muscle strength and tone were normal in all 4 limbs. Serum creatine kinase (CK) level was 2280 IU/L (normal value < 200) while liver enzyme levels were slightly increased, including aspartate aminotransferase (AST) (120 IU/L; normal value <40 IU/L) and alanine aminotransferase (ALT) (57 IU/L; normal value <40 IU/L). Serum antinuclear autoantibodies/extractable nuclear antigens (ANA/ENA) levels were normal. Electrocardiogram and D-dimer level were normal. The B-type natriuretic peptide (BNP) level was 606 pg/mL and the level of soluble growth stimulating gene protein (sST2) was 97.64 ng/mL (normal value <35 ng/ml). Findings of arterial blood gas (ABG) analysis were: pH 7.286; PaCO2 73.6 mmHg; PaO2 62.7 mmHg; lactate 2.82 mmol/L (normal value <2.2 mmol/L); hemoglobin 177g/L. Although the PaO2 level was within the normal range on low-flow oxygen inhalation, the diagnosis of type II respiratory failure was considered because of hypercapnia. Pulmonary function test suggested severe restrictive ventilatory impairment. Chest high resolution CT showed bilateral pleural effusion (more on the right side) and increased thickness of pulmonary artery trunk and branches. The patient showed none of emboli, mosaic perfusion, disparity in segmental vessel size, parenchymal densities and thickening of bilateral pulmonary arteries in Computed tomographic pulmonary angiography (CTPA). Echocardiography revealed right heart overload characterized by dilated right ventricle (RV diastolic dimension: 44 mm) and right atrium (RA dimension: 45 mm) (Fig. 2). Color Doppler ultrasound revealed mild tricuspid regurgitation and mild to moderate pulmonary hypertension; the systolic pulmonary arterial pressure was 56 mmHg. The tricuspid annular plane systolic excursion (TAPSE) level was 22 mm. Echocardiography showed normal left ventricular chamber volume with normal systolic function (estimated ejection fraction 71%) and normal filling pressures. Left ventricle short-axial view showed symmetric slight thickening of the left ventricle (LV) and the LV mass indexed for BSA was 118.07g/m2. At the end diastolic phase of the left ventricle, the thickness of both the ventricular septum and the posterior left ventricular wall is 13 mm.

Needle electromyography (EMG) examination showed rapid recruitment of short-duration, low-amplitude motor unit potentials (MUPs) in bilateral deltoid muscles. He underwent muscle biopsy of left lateral thigh, which revealed variable size and shape of muscle fibers, with coexistence of oblong or small round fibers, occasional necrosis or regeneration of muscle fibers, hypertrophic fibers and split fibers in addition to typical features of mitochondrial dysfunction, including 8% ragged red fibers (RRF) and 42% COX-negative fibers.

**Fig. 3:**
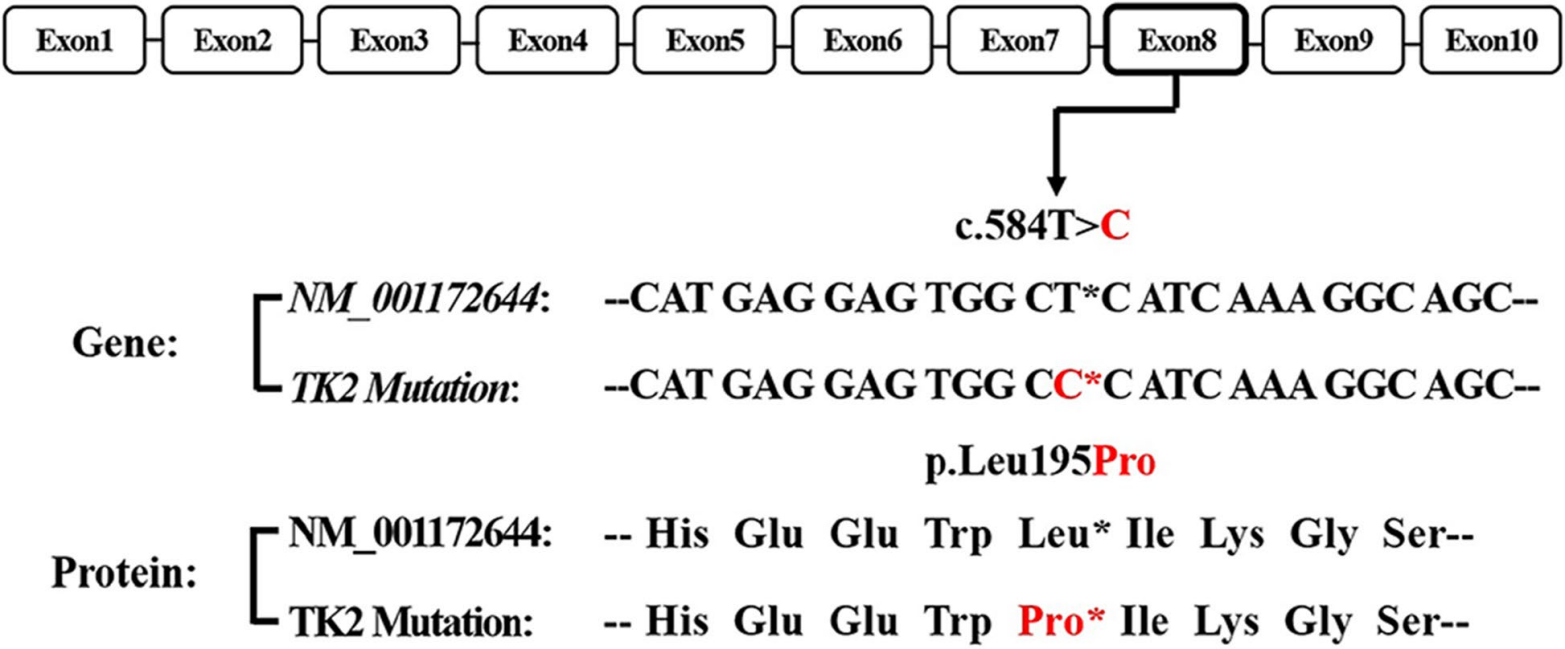
A novel *TK2* variant (NM_001172644: c.584T>C, p.Leu195Pro) was discovered in a patient with mitochondrial myopathy. Single nucleotide mutation detected by second generation gene sequencing

Gene sequencing analysis conducted by BGI Clinical Laboratory Center (Shenzhen, China) revealed a novel *TK2* mutation at c.584T>C. The predicted amino acid change was p. Leu195 Pro. Whole exon gene results of the proband and family members showed that the mother had wild-type and the father, sister, and the patient had heterozygote *TK2* variant (NM_001172644: c.584T>C, p.Leu195Pro). In-depth analysis revealed a heterozygous variant in an intron of *TK2* (NM_004614.4:c.156+958G>A; rs1965661603) in the mother of the proband, the sister, and the proband. Based on the findings of muscle biopsy and DNA sequencing, the patient was diagnosed as a case of MM (Fig. 3).

**Fig. 4:**
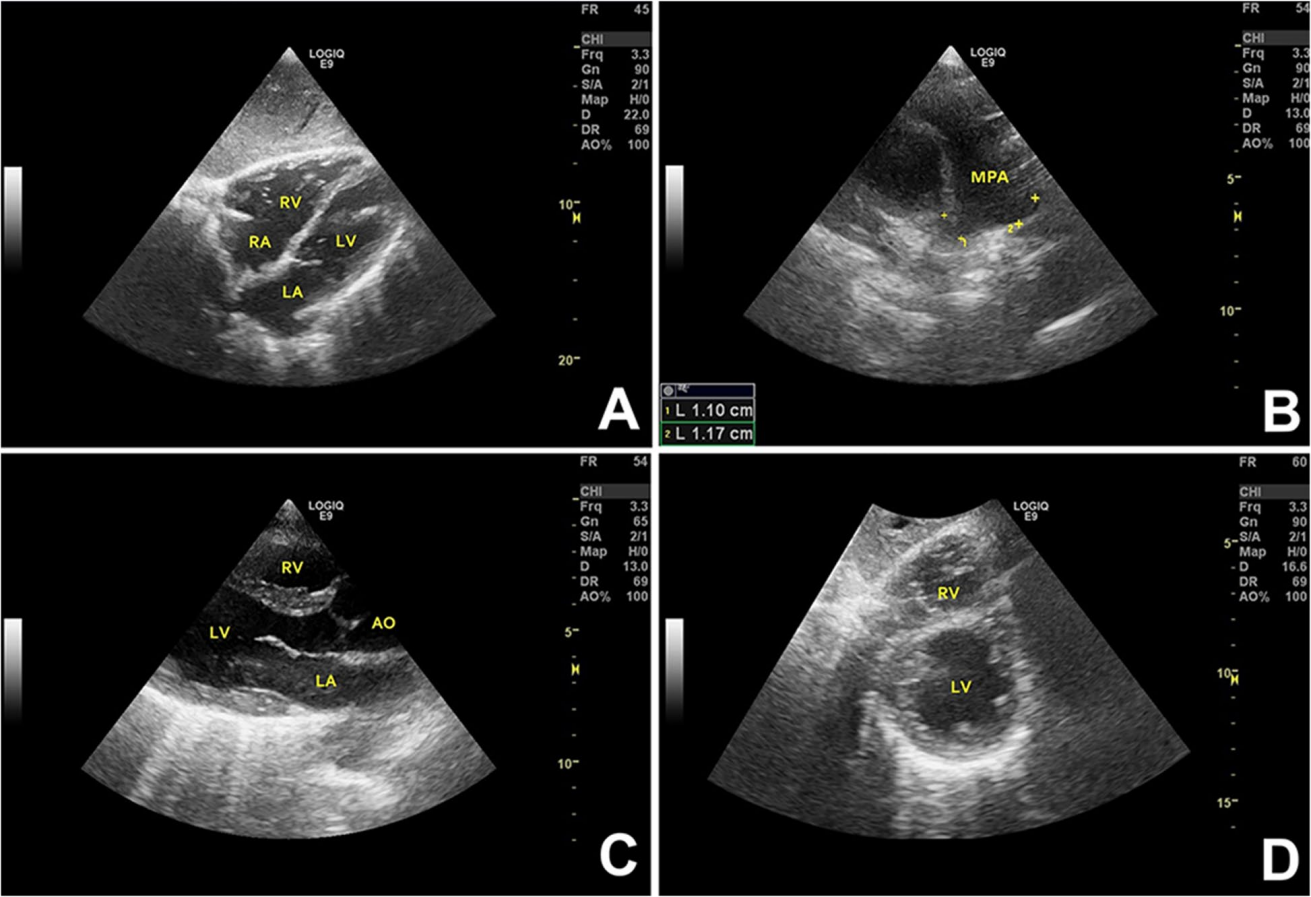
Echocardiography of the patient after one month of treatment (**A-D**). Apical four-chamber view showing normal RA and RV (**A**); Short-axis view of the heart showing the normal main pulmonary artery (MPA) and its branches (**B**); Long axial section of LV showing the normal range of left atrial (LA) and LV size (**C**); Left ventricle short-axial view showing normal chamber wall thickness; LV end-diastolic wall thickness is 9 mm (**D**)

He was administered intravenous diuretic and recombinant human brain natriuretic peptide. Round-the-clock non-invasive ventilation was provided during the first 10 days of hospitalization; subsequently, artificial ventilation was provided only at night. The shortness of breath was significantly alleviated with complete resolution of bilateral lower limb edema. ABG analysis results prior to discharge were: pH 7.388; PaCO_2_ 52.7 mmHg; PaO_2_ 123.5 mmHg; and hemoglobin 155 g/L. The patient was prescribed with the nocturnal non-invasive ventilation and coenzyme Q10 (CoQ10) at discharge. CoQ10 can promote oxidative phosphorylation and protect the integrity of biological cell membrane, it increases adenosine triphosphate (ATP) generation and cellular energy by mediating electron transfer in the electron transport chain. CoQ10 can be used as an adjunctive therapy for mitochondrial myopathy or heart failure. Echocardiography performed after one month showed no obvious abnormalities in cardiac structure or function. Echocardiography findings also showed improvement after treatment (Fig. 4). No abnormalities were observed in BNP and sST2 levels. CK was reduced to 1250 IU/L and liver enzyme levels were restored to normal. ABG parameters were within the normal range (pH 7.411; PaCO_2_ 42.9 mmHg; PaO_2_ 86.5 mmHg; lactate 2.0 mmol/L; hemoglobin 151 g/L). A one-year follow-up, the patient was found to have well tolerated non-invasive ventilation support. He was able to perform his routine activities with no restrictions. Echocardiography showed normal cardiac structure and function. Currently, nocturnal bilevel positive airway pressure (BiPAP) therapy via a basal mask had been well-tolerated and ABG analysis was performed every 1 month, the ventilation pattern had not been changed after discharge. The patient is regularly receiving CoQ10.

## Discussion and conclusions

Here we report a case of MM which presented with coexistence of acute respiratory failure (ARF) and acute heart failure (AHF). To the best of our knowledge, this is the first reported case of reversible cardiac structural and functional changes, especially, reversible left ventricular hypertrophy.

Currently, there is a paucity of literature on adult-onset MM presenting with ARF and AHF. Some studies have reported a small proportion of patients, mostly children and rarely adults, showing both respiratory and cardiac dysfunction [6–8]. In a recent study of 21 pediatric cases of MM, 3 patients (14.3%) presented with sudden respiratory failure, and 5 showed cardiac enlargement, tricuspid incompetence, and pulmonary artery hypertension on echocardiography. However, this study did not report the proportion of patients with simultaneous respiratory failure and heart failure [9]. In a recent report by Cristina et al, 2 out of 18 patients with *TK2* mutation-associated MM had respiratory insufficiency as the first symptom [10]. In this series, all patients showed respiratory muscle weakness, and 12 patients required non-invasive mechanical ventilation. However, there were no data on cardiac structural and functional abnormalities. The present case of MM presenting with ARF and AHF as initial symptoms fills a gap in the contemporary literature. The patient presented with type II respiratory failure as the predominant clinical manifestation on his admission. Pulmonary function test suggested severe restrictive ventilatory impairment.Weakness of the primary inspiratory muscles or dysfunction of the respiratory centers in the brain may have contributed to ARF in the patient.

Our patient who presented with ARF and AHF resumed normal daily life after 1-month non-invasive ventilation and medication. His cardiac structure and function had also returned to normal at 1 month. His echocardiographic measures were normal at one-year follow-up. From a pathophysiological point of view, respiratory muscle dysfunction results in hypoxia and respiratory failure, which causes increased pulmonary artery resistance, pulmonary artery hypertension, right heart enlargement, tricuspid valve insufficiency and regurgitation [11, 12]. With the improvement of respiratory function, there was complete recovery of the structure and function of the right heart, suggesting that the right heart changes were a result of pulmonary artery hypertension caused by respiratory muscles weakness rather than mitochondrial cardiomyopathy. Combined with history, clinical evaluation, including pulmonary function tests, chest high resolution CT, echocardiography, CTPA, and serology of ENA and follow-up findings, we concluded that this patient had type 3 pulmonary hypertension due to hypoxia. Unfortunately, the patient refused to have a right heart catheterization done during this hospitalization, and we were unable to obtain more detailed pressure data, such as pulmonary vascular resistance and pulmonary arterial wedge pressure.

To the best of our knowledge, this is the first report describing left ventricular hypertrophy in a patient with MM presenting with respiratory failure and right-sided cardiac insufficiency. It is also the first case study to explore the association between left ventricular hypertrophy and hypoxemic respiratory failure in a patient with MM.

The correlation between hypoxia and left ventricular hypertrophy warrants further investigation. Recent studies have found a correlation of left ventricular mass (LVM) and thickness with nocturnal hypoxemia in different diseases. For example, in a study, nocturnal hypoxemia was found to be an independent predictor of LVM and thickness. Nocturnal O_2_ desaturation has been linked to left ventricular hypertrophy in hemodialysis patients, and this association was found to be largely independent of arterial pressure [13]. In addition, intermittent hypoxia was shown to be significantly associated with higher values of LVM and LVM index (LVMI) in patients with obstructive sleep apnea-hypopnea syndrome [14]. Moreover, in a study of patients with chronic obstructive pulmonary disease, those with significant nocturnal hypoxemia had increased LVM compared with those with mild daytime hypoxemia [15]. These findings indicate that LV hypertrophy occurs in patients with nocturnal hypoxemia.

In this patient, echocardiography showed left ventricular hypertrophy on admission. Left ventricular wall thickness was normal one month after therapy. Since the patient had no history of systemic hypertension, or ischemic or valvular heart disease, the changes in structure and function of the RV and LV were considered to be adaptive responses to respiratory failure caused by mitochondrial myopathy. Before the onset of clinical symptoms of right heart failure, the right ventricle has developed concentric hypertrophy in order to adapt to changes in pulmonary artery pressure, and the appearance of symptoms may represent a development of eccentric hypertrophy of the right ventricle. Animal studies had shown that LV hyperthrophy was associated with the duration of right ventricular (RV) pressure overload [16]. Although the patient did not undergo sleep monitoring after discharge, the treatment outcome showed that wearing artificial ventilator only at night can reverse ventricular hypertrophy. The altered structure of the RV and LV might be attributed to the intermittent increases in hypoxia that most likely occur during sleep. Although impairment of the structure and function of the right heart is considered as the primary adverse effect of respiratory failure in patients with MM, we argue that perhaps more attention should be given to the impact of respiratory disease on the left ventricle, since dysfunction of the left ventricle is significantly associated with life-threatening events.

Despite the restoration of the patient's respiratory and cardiac function at 1-month follow-up under the treatments of non-invasive ventilation and CoQ10, there were no disease-modifying treatments available for this patient. The treatment was aimed at improving the quality of life of the patient. Hernandez-Voth recently reported the results of a study in which deoxythymidine and deoxycytidine were orally administered to 6 adult patients with thymidine kinase 2-deficient myopathy [17]. Deoxynucleoside therapy was effective in improving respiratory function and diaphragmatic function in the short-term and in stabilizing the loss of respiratory capacity in the medium-term( 17). Deoxynucleoside treatment may be a promising treatment for our patient.

It should be highlighted that echocardiography and laboratory tests in our case captured the reversible cardiac structural and functional changes before and after treatment, which has not been previously reported in patients with MM. This is the first report to suggest that nocturnal hypoxia is a potential mechanism for left ventricular hypertrophy in patients with MM. Moreover, early management of the respiratory function can improve the patient's prognosis.

A limitation of this case report is that we could not identify the novel *TK2* mutation as the disease-causing gene based on current genetic test results. More functional tests (such as “rescue” experiments) may help confirm the pathogenicity of the mutation.

In conclusion, MM is a rare myopathy disorder characterized by mild muscle weakness of the proximal limbs, chronic and progressive external ophthalmoplegia, and slow progression of respiratory dysfunction. This disease can not only lead to respiratory failure but also structural changes in the heart, resulting in left ventricular hypertrophy and cardiac dysfunction. Nocturnal hypoxia is a potential mechanism for left ventricular hypertrophy in patients with MM. Early diagnosis and management of patients with MM can significantly ameliorate the life-threatening respiratory and heart failure events related to the disease. Our patient presented with skeletal muscle weakness, ptosis, fatigue, and exercise intolerance that progressively worsens over time. In addition, this patient presented with slowly progressive peripheral muscle weakness and respiratory failure requiring mechanical ventilation. As reported in this case, early diagnosis and long-term non-invasive ventilation support enabled the patient to fully recover and return to work and normal daily life.

## Data Availability

The raw data presented in the study are included in the article. Further inquiries can be directed to the corresponding authors.

## Abbreviations

MMs: Mitochondrial myopathies
MM: Mitochondrial myopathy
mtDNA: Mitochondrial DNA
nDNA: Nuclear DNA
RRF: Ragged red fibers
CoQ10: Coenzyme Q10
CK: Creatine kinase
AST: Aspartate aminotransferase
ALT: Alanine aminotransferase
BNP: B-type natriuretic peptide
sST2: soluble growth stimulating gene protein
ABG: Arterial blood gas
TAPSE: Tricuspid annular plane systolic excursion
RV: Right ventricle
RA: Right atrium
LV: Left ventricle
LA: Left atrial
MUPs: Motor unit potentials
ARF: Acute respiratory failure
AHF: Acute heart failure
LVM: Left ventricular mass
LVMI: Left ventricular mass index
CTPA: Computed tomographic pulmonary angiography
